# Intelligent recommendation system based on decision model of archive translation tasks

**DOI:** 10.3389/fncom.2022.1048047

**Published:** 2022-11-04

**Authors:** Chen Lilan, Chen Yongsheng

**Affiliations:** ^1^School of Foreign Languages, Guangdong Pharmaceutical University, Guangzhou, China; ^2^School of Information Management, Sun Yat-sen University, Guangzhou, China

**Keywords:** intelligent recommender system, multi-label classification, decision model, classification algorithm, adaptive

## Abstract

How to recruit, test, and train the intelligent recommendation system users, and how to assign the archive translation tasks to all intelligent recommendation system users according to the intelligent matching principles are still a problem that needs to be solved. With the help of proper names and terms in China’s Imperial Maritime Customs archives, this manuscript aims to solve the problem. When the corresponding translation, domain or attributes of a proper name or term is known, it will be easier for some archive translation tasks to be completed, and the adaptive archive intelligent recommendation system will also improve the efficiency of intelligent recommendation quality of archive translation tasks. These related domains or attributes are different labels of these archives. To put it simply, multi-label classification means that the same instance can have multiple labels or be labelled into multiple categories, which is called multi-label classification. With the multi-label classification, archives can be classified into different categories, such as the trade archives, preventive archives, personnel archives, etc. The system users are divided into different professional domains by some tests, for instance, system users who are good at economic knowledge and users who have higher language skills. With these labels, the intelligent recommendation system can make the intelligent match between the archives and system users, so as to improve the efficiency and quality of intelligent archive translation tasks. In this manuscript, through multi-label classification, the intelligent recommendation system can realize the intelligent allocation of archive translation tasks to the system users. The intelligent allocation is realized through the construction of intelligent control model, and verifies that the intelligent recommendation system can improve the performance of task allocation over time without the participation of task issuers.

## Introduction

Only relying on human resources to classify large-scale archive sets is faced with great challenges. There are a large number of China’s Imperial Maritime Customs archives, for instance, there are 16,115 volumes of modern Guangdong Customs archives stored in Guangdong Provincial Archives. And there are even more related archive materials, including the original archives, Customs publications, Chinese staff publications, physical archives, etc. According to the types of archives, China’s Imperial Maritime Customs archives can be divided into Circulars and S/O Circulars issued by the Inspectorate General of Maritime Customs to Customs Stations; the Despatches and S/O Letters between Inspector General and Commissioners; the Printed Notes/Circular Memorandums issued by the Deputy Inspector General to Customs Stations; the Memorandums issued by Departments of Inspectorate General of Maritime Customs to Customs Stations; Telegrams, Service List, Local Rumours, Documents, Files and Accounts, etc. From the perspective of the issuing institutions, China’s Imperial Maritime Customs archives can be divided into Tax Archives, Preventive Archives, Personnel Archives, Secretary Archives, Archives of General Affairs, Marine Archives and Postal Archives, and so on. According to the domains involved, these archives can be divided into economic archives, personnel archives, trade archives, language archives, etc. In order to sort out and classify these digital archives with multi-labels, we need to find a method with high efficiency and low cost.

The paper “Spatial-Temporal Adaptive Intelligent Allocation of Archival Tasks” (Lilan and Yongsheng, 2021) describes the method of allocating the archive translation tasks on the adaptive intelligent recommendation system to all available system users based on language barriers. This manuscript attempts to realize the intelligent allocation between the system users and China’s Imperial Maritime Customs archives from the perspective of multi-label classification and classification construction on the basis of using the proper names and terms in China’s Imperial Maritime Customs archives. However, as analysed above, there are different types and various forms of China’s Imperial Maritime Customs archives, and different professional knowledge backgrounds and different skill levels of the adaptive intelligent recommendation system users (Lo and Fujiwara, 1996; Wang et al., 2019; Wang et al., 2021). Therefore, when allocating these archive translation tasks to the system users, how to allocate different types of archives to users with different professional backgrounds, so as to achieve the intelligent matching between the archives to be translated and the system users, save labour force, and improve the quality and efficiency of archive translation tasks? (Paul and Harrison, 2005). This is a problem that needs to be prioritized and addressed.

After selecting, testing and training the adaptive intelligent recommendation system users, based on the previous experiment of allocating the archive translation tasks to all the available system users, this manuscript puts forward a classification method by asking the system users simple questions about the professional knowledge of Chin’s Imperial Maritime Customs archives, classifying these users into different backgrounds, such as economic and financial, laws, labour force management, local histories, linguistics, etc. Then, according to such characteristics as archive issuing institutions, archive titles, archive types, we comprehensively consider and construct the multi-label classification characteristics of the archives, so as to realize the intelligent allocation between the archives and the system users.

The so-called distributed intelligent recommendation system proposed recently classifies the system users by asking them simple questions. However, the distributed intelligent recommendation system is not intelligent. Considering the cost, the total cost of a classification generated by the distributed intelligent recommendation system is almost the same as that generated by an expert. However, the labour force required by the distributed intelligent recommendation system is six times that required in the classification construction by experts, which indicates that the distributed intelligent recommendation system is expensive in labour force and cost. So how to improve the workflow and make the classification process cheaper and more efficient?

Therefore, this manuscript tries to improve the distributed intelligent recommendation system with the adaptive method proposed previously (Leong and Yen, 2008; Ross, 2014). With the workflow and category label of distributed intelligent recommendation system, the adaptive method allocates archives to the system users, which saves cost and improves efficiency. In the case of a large amount of labour force generated by each archive-label pair in the distributed intelligent recommendation system, the adaptive method can save labour by using the labels and the learning model of co-occurrence probability (Wang et al., 2019) to sort out archive tasks intelligently.

This manuscript has the following innovations:

• This manuscript proposes an effective solution to the problem of multi-label classification, and describes the decision-making theory including the following two parts: (1) the probability model in which the system users estimate the truth value of the archive-label relationship according to a certain probability precision, and (2) the controller which dominates archive translation answers to each archive document to be translated so as to provide the greatest value for the joint classification.
• This manuscript theoretically verifies the efficiency of the control strategy, and also provides an effective method to select batch labels, so as to ensure the universality of the method proposed in this manuscript for the system.
• In this manuscript, experiments are carried out on the adaptive intelligent recommendation system, and the results show that the intelligent strategy of the adaptive intelligent recommendation system needs less than 10% of the labour force required in the distributed intelligent recommendation system.

In addition to reducing the cost of multi-label classification and classification construction, the adaptive experiments also show that compared with the previous simple task-based workflow, artificial intelligence and decision-making theory can be applied to more complex workflow (Nemhauser et al., 1978; Krause and Guestrin, 2005; Chilton et al., 2013; Sun, 2017).

## The basic classification algorithm

Both the distributed intelligent recommendation system and the improved adaptive intelligent recommendation system in this manuscript need to input a group of archives to be classified, such as photos or text fragments. Their output is a tree-like structure, whose internal nodes are marked with text string labels (types).

**FIGURE 1:**
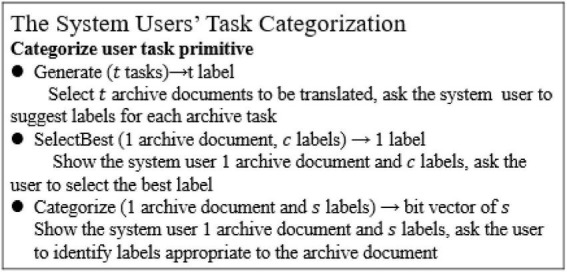
Sample of the system users’ task classification in experiments.

The classification construction algorithm in this manuscript takes a series of algorithm steps and three task options, and asks the system users to create labels step by step. From a functional perspective, these tasks can be described in Figure 1.

This manuscript seeks to minimize the number of tasks (tasks here refer to archive documents to be translated) for the system users to solve the efficiency problem of multi-label classification. Both the distributed intelligent recommendation system and the adaptive intelligent recommendation system start from “Generate” step to brainstorm and generate a set of candidate category labels. They take the “SelectBest” step to filter out the undesirable labels, and “Categorize” step to select the appropriate category labels for all archive documents to be translated. When most of the archive documents corresponding to one label are contained in the archive document set corresponding to another label, a hierarchical structure is constructed from the data by introducing a parent-child relationship between the two labels; labels with few corresponding archive documents are deleted and labels overlapping with too many other labels are merged. This is the global structure inference.

**FIGURE 2:**
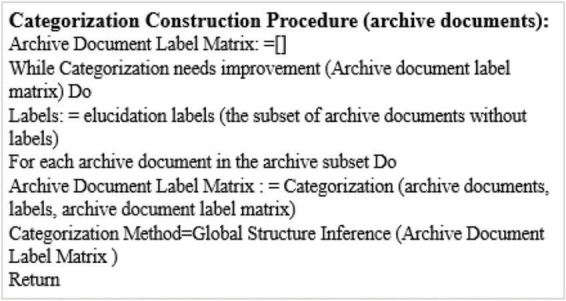
The construction of classification algorithm.

Figure 2 shows the classification construction procedure. The distributed archive allocation and the adaptive archive allocation are completely different in terms of termination conditions, label elucidation and classification actions. However, the figure does not specify how the adaptive archive allocation algorithm elucidates the classification label set, nor how to effectively classify each archive document with the fixed label set. These problems will be discussed in the next two sections. In short, the distributed intelligent recommendation system takes a relatively simple method for these tasks, but the more intelligent and comprehensive adaptive intelligent recommendation system can greatly reduce the labour force required.

Here, taking the classification labels of the archive documents in the system as an example, the test questions for archive classification are designed as follows:

1. The language features of official documents are ______? (multiple choices)
  □ simple and formal □ with complex sentence structures
  □  with many long sentences  □  with many short sentences
2. What are the characteristics of the format for official documents? (multiple choices)
  □ The title should indicate the type, the cause and the issuing institution of the document.
  □ There should be the issuing time of the document.
  □ There should be a salutation.
  □ The title should conform to the recipient(s) of the document.
  □ The signature should be provided.
  □ The format of the official document in both Chinese and English are the same.
3. In the archive segment “…I.G. Circular No. 2654 directs that the products of the Hua Ch’ang De Chi Cloth Factory, of Shanghai, are to be added to the list of Chinese factory products to which the single duty payment privilege has been accorded.” The Chinese name “华昌德记布厂” is spelt as “the Hua Ch’ang De Chi Cloth Factory,” in which spelling method is it spelt?
  □  Nicolas Trigault spelling □ Matteo Ricci’s spelling
  □ Wade–Gile’s spelling □ Modern Chinese Pinyin system
4. What is the corresponding Chinese counterpart of the position “Assistant Advisor”?
  □ 验估员 □ 副验估员 □ 监察员
  □ 副监察员 □ 副验船员
5. The Chinese organization corresponding to “Chief Secretary” is________.
  □ 总务科 □ 秘书科
  □ 总秘书 □ 汉文科
6. Which place does “towmoon” refer to in Chinese?
  □ 福州的陡门 □ 杭州的斗门 □ 拱北的斗门
7. In “…I have now to circulate, for your information and guidance, copy of Shui-wu Ch’u despatch No. 155, laying down exactly which provincial Huchao may be recognized in this connexion. Huchao ciocced by Jufa (since abolished), Chiang-chün (将军), Ju-t’ung (都统), Ganieon Commicoioner (镇守使), and Defense Commicoimers (护军使).” issued on July 22nd, 1914, which word implies the archive type of this official document?
  □ Shui-wu Ch’u □ despatch
  □ circulate □ information
8. What type of archives does the archive segment “…This dispatch will be handed to you by Mr. Au Yuk-shing, Assistant Examiner B, granted 3 months’ extension of sick leave, without pay, from 1st March to the 31st May 1935, by I. G. dispatch No. 2834/156011 to Ningpo, copy of which has been sent to you….” belong to?
  □ Personnel □ Secretary
  □ Financial □ Service list
9. “…I beg to inform you that I am to-day forwarding to your address by Tow “Man Tsu” (民族) one box containing one package of Native Raw Opium weighing 4.5 Hectogrammes under Kongmoon S/R. No. 338.
  I would request you to be good enough as to take charge of this seizure and to destroy same at your next burning of opium, etc., in the presence of the Superintendent’s representative. …” In this archive segment, what was the recipient required to do?
  □ to take charge of and destroy the opium seized
  □ to seize the opium □ to inform the seizure of opium
10. How many grams of opium are reported in the above archive segment?
  □ 4.5 hg □ 450 g
  □ 4.5 kg □ 4500 g
11. In the archive segment “…I beg to enclose a cheque for Hkg. 23.48 representing the Tonnage Dues and Surtax collected at Lappa during the September Quarter, 1935. These Dues were collected from 13 launches of which:
  10 were under the Chinese flag
  and paid $ 20.29 = Hkg. $ 15.02
  3 were under the Portuguese
  flag and paid $ 5.25 = 3.93
  18.95
  30% Surtax on launches under
  Chinese flag $ 5.75 = 4.53
  Hkg. $ 23.48”
  Which currencies’ exchange is described?
  □ USD and HKD □ State Currency and HKD
  □ USD and Haikwan Tael
  □ State Currency and Haikwan Tael
12. In the archive segment “…Mr. Poletti’s pay, Expatriation Allowance and G. U. Pay Adjustment and Actg. Allce. (1st—30th: $77.90) have been issued to him to the 30th September 1935 and $2+\frac{1}{2}$ passages have been provided for himself, family and—to Shanghai together with a mileage allowance ($229.00) to Canton. …” what do “Expatriation Allowance” and “G. U. Pay Adjustment” represent in Chinese, respectively?
  □ 调岗津贴和薪酬调整
  □ 移民补贴和薪酬调整
  □ 调职津贴和黄金薪酬调整
13. In the Circular segment “…I enclose, in Chinese, three Rules that have been approved of by H.E. the Acting Imperial Commissioner Li, affecting goods passing the Barriers nearest the port, when being conveyed to or from the interior.” issued by the Inspectorate General of Maritime Customs on April 18, 1863, what does “H.E.” represent? And who did the “Acting Imperial Commissioner Li” refer to in Chinese?
  □ H. E. represents “He”
  □ H. E. represents “Your Majesty”
  □ H. E. represents the Emperor
  □ “Acting Imperial Commissioner Li” refers to 李莲英
  □ “Acting Imperial Commissioner Li” refers to 李鸿章
14. What should be paid attention to when translating the above archive segment? (Multiple choices)
  □ to understand correctly □ to express exactly
  □ the appropriate translation method, such as addition
  □ the diction is appropriate to the background in the original archive document
15. In the archive segment “…The junks, laden with foreign goods from Kwangchowwan, were bound for Shang Tsun Chai (上村仔) [Cho Soan Chi (上村仔) Village: Appendix Memorandum], in the vicinity of Pak Shek Chai (北石仔), on the Luichow (雷州) Peninsula south of Malomoon (马罗门). …” in which spelling method were these place names spelt?
  □ the Wade-Gile’s spelling □ the Postal spelling
  □ the Postal + Dialect □ the Modern Chinese Pinyin System
16. Which bank does “the Oriental Bank Corporation” refer to in Chinese in the archive segment “…The balance in hand at the end of each quarter, you will have the goodness, unless otherwise instructed, to remit to the Oriental Bank Corporation [Shanghai or Hongkong] to be placed to the credit of my Account B”?
  □ 东方银行 □ 丽如银行 □ 汇丰银行 □ 中央银行
17. In the archive segment “…With reference to your despatch No. 1,287/Kowloon: forwarding, at my request, demand drafts for Hkg. $ 933.86 and Hkg. $ 786.08, in settlement of Seizure Rewards for salt; …” what does the “demand drafts” refer to in Chinese, and which domain does it belong to?
  □ 即期汇票, international trade □ 即期汇票, economy
  □ 远期汇票, economy □ 草稿, painting
18. Which type of archives does the archive segment “…This despatch will be handed to you by Mr. Au Yuk-shing, Assistant Examiner B, granted 3 months’ extension of sick leave, without pay, from 1st March to the 31st May 1935, by I. G. despatch No. 2834/156011 to Ningpo, copy of which has been sent to you. …” belong to according to the types and issuing institution?
  □ despatch from the Personnel Department
  □ despatch from the Secretary Department
  □ despatch from the Department of General Affairs
19. In the archive segment “…This despatch will be handed to you by Mr. Chung Kwei Hsin, Probationary Tidewaiter, transferred to your port by Inspectorate despatch received on the 11th November, 1935. …” what position does the “Probationary Tidewaiter” refer to in Chinese?
  □ 试用钤子手(1927年前)/试用稽查员(1927–1947)
  □ 试用帮办 □ 候补守备
  □ 试用头等总巡 □ 总司录事
20. What does the archive segment describe in “An anonymous letter to Mr. Yang Ming Hsin, Commissioner of Kongmoon Customs, dated 10th December 1937. Stating that the Yung Yung (Yungki) Customs and MR. Ip Yau Cheong, the Samshui Tidesurveyor, cooperating with the crew of Wuchow steamers especially the s.s. Chung On, are engaged in smuggling; that sharks’ fins, birds’ nests and other sundries are being concealed in an ice chest at the bow; while canned goods, sugar, and sundries are being concealed in sofa, wooden cases, etc., in the dining room, first class cabins, store rooms, and boys’ rooms; and that goods to the value of $500/600 are smuggled in every trip”?
  □ Customs Preventive □ Customs staff smuggling
  □ Customs staff cooperation

By judging which categories a certain archive document belongs to, the upward, downward, or parallel; the personnel, preventive, or notice; business, or administrative; and other types, whether it be reported in the late Qing Dynasty or the Public of China, it’s further determined a specific domain among economic, financial, language, personnel, and other specific domains. By constructing the corresponding relationship between these labels and archives, when allocating archive translation tasks with the adaptive method, it can be more targeted to achieve the intelligent allocation between archives to be translated and the system users. The classification of the system users is similar to the classification of archives. The following two sections describe the elucidation and classification of category labels of archives.

### Elucidation of category labels

First of all, let’s take a look at the elucidation steps of the category labels in the distributed intelligent recommendation system. If the system users are required to brainstorm candidate labels for each archive document through the “Generate” step, it may cause label duplication. The distributed intelligent recommendation system only considers the first few (m=32) archive documents when executing the label elucidation task, which is called the initial archive set. The distributed intelligent recommendation system divides the initial archive set into *t*=8 groups, and constructs a “Generate” step for each group, which is sent to *k*=5 system users. After completing all the [*km*/*t*] tasks, the distributed intelligent recommendation system will leave *km* candidate labels.

Next, the distributed intelligent recommendation system will delete some candidate labels. Now each of the *m* initial archive documents has up to *k* different labels. For each archive document, the distributed intelligent recommendation system submits *k* “SelectBest” steps to let the system users choose which labels are the best.

In the next section, we will use the combinatorial model to describe the decision-making approach to monitoring label elucidation.

### Classification of archives when the label is known

After the elucidation of the category labels, the distributed intelligent recommendation system will enter the next stage, which will bring *O*(*np*) tasks to the system users, where *n*=|*archivedocument*| and *p*=|*label*|. To put it simply, this is to iterate the archives and labels, asking *h* different system users whether a label is applicable to an archive document. Chilton and Schäffner (2002) observed that it’s difficult for some system users to make a choice due to the lack of context. Therefore, they proposed two sequential stages for classification, which is called adaptive filtering. In the first stage, the archives and labels are iterated in the above way; the labels which obtain at least two votes among five enter the next stage. In the second stage, the system users can only see the labels after the first round of deletion. If at least four of the five system users think that the label is suitable for an archive document, the label is considered to be suitable for the archive document.

This manuscript proposes several improved algorithms for this classification process in two parts, which is a multi-label classification issue. The labels generated by the first method are identical, and the tasks for the system users are fewer; the labour force required by the second method is greatly reduced, and the classification accuracy is almost not affected; finally, the probability model of label generation and concurrency is gradually constructed to optimize the order of allocating archive translation tasks to the system users.

## Polya urn model for label elucidation

The label elucidation step requires the adaptive intelligent recommendation system users to brainstorm and add relevant labels to the classification. This manuscript first performs this step on a group of *m* files, where *m*≪*n*, *n* is the total number of archive documents to be classified. When the number of archive documents that need label elucidation is small, because the labels generated by random subset of archive documents are globally related, and the system users may repeat labels for archives, the related labels can be added to the classification. One of the key control problems in optimizing this step is the selection of *m*, which is set as *m*=32. But ideally, this manuscript hopes to estimate the performance when the classification label set is expanded, so as to determine the time to terminate the label elucidation.

In this manuscript, the Polya urn model, which is applied in the adaptive system, is used to model the label elucidation process, also known as the Chinese restaurant process. The Polya urn model is particularly suitable for modelling discrete, multi-label distribution, in which the number of labels is unknown in advance. This model can be compared to an urn with coloured balls, in which the colours correspond to the labels. In each iteration, a ball is evenly extracted from the urn and then put back into the urn with a new ball. If the extracted ball is black (a specially specified colour), the colour of the new ball is not seen before; otherwise, the colour of the new ball is the same as that of the extracted ball.

When the ball is removed from the urn, the number of colours in the urn increases, but the probability of obtaining new colours decreases. In addition, the colour extracted more frequently has higher extraction probability than other colours. This phenomenon can be seen from the probability which dominates taking balls from the urn. Suppose there are *N* non-black balls, *n_c_* balls in specific (non-black) colour *c*, *a* black balls. Then, the probability of taking out the ball of colour *c* is *n*_*c*_/(*N* + *a*) and the probability of taking out the ball without seeing the color before is *a*/(*N* + *a*). The Polya urn model is parameterized by *a*; the larger the value of *a*, the greater the probability of brainstorming a new category label.

### Theorem 1

Let Polya urn model contain *N* colored balls and *a* black balls. Let the random variable *X_d_* be the number of new coloured balls in the urn after *d* times of extraction in the future, then


$$E\,\left[X_{d}\right]=\sum_{i=0}^{d-1}\frac{\alpha }{N+\alpha +i}$$

In this manuscript, *k* system users brainstorm labels for each archive document. If labels have been generated for *m* archive documents and *n* − *m* = *r* archive documents are left, then *N* = *km*, *d* = *kr*. At this time, if the label elucidation phase is terminated, the expected labels of $\sum_{i=0}^{k\,r-1}$^α^/_(km+α+i)_ number will be lost, and the expected increase of the total number of labels is the different number of labels obtained by dividing this number by the *m^th^* archive document. Detailed proved in Mahmoud (2008).

The model in this manuscript provides a stop condition for the label elucidation phase: when the expected small-scale increase of the number of labels is lower than the expected threshold, the phase ends. In order to implement this strategy, we use the log likelihood gradient of the generated observed data to calculate the maximum likelihood estimation of *a*. Assume that all labels in this model are independent, and the system users can generate new labels for any specific archive document.

## Improved classification control algorithm

Like the workflow of many adaptive allocation systems, this manuscript requests a fixed number of votes *k* and sets the threshold of vote *T* (most votes are special cases, where *T* = *k*/2) to implement binary votes. This process returns to T if and only if the affirmative vote is at least *T*. This process requires a lot of work. In the adaptive filtering step of the workflow, the distributed intelligent recommendation system requires *k* system users to vote on the combination of each archive document and labels. Suppose there are *n* archive documents and *p* labels, then this process requires *O*(*knp*) votes.

### Lossless improvement of threshold voting

The first phenomenon observed in this manuscript is that when a threshold of vote *T* is given, if *T* positive votes or (*k* − *T* + 1) negative votes have been collected, because the result of using the total number of *k* votes is completely positive in the former case and negative in the latter case, no further voting is needed. In this manuscript, we call this stop condition lossless stop, which can be regarded as a summary of the strategy of “asking two people to vote, if the two people disagree, asking the third person to vote.”

### One-way heuristic threshold voting

A simple heuristic method can further reduce the number of votes needed, which is called one-way heuristic voting in this manuscript. Compared with the original threshold voting method, this method leads to fewer errors. If max{*T*−1,0} positive votes are observed with no negative votes, the heuristic will return T in advance; if max{*k*−*T*, 1} negative votes are observed with no positive votes, it will return to F in advance.

### Bayesian probability model

Suppose that the labels of a large number of archive documents in *I* ∈ 𝒥 have been given. For each archive document *I*, if the label *L*=*yes*, then it is expressed as ⊕(*I*, *L*)=1; if stop, then it is expressed as ⊕(*I*, *L*)=0. When a new archive document *I*′ is given, in this manuscript we will use the previously observed data to calculate the largest likelihood preteriori probability of any label $P\left[⊕\left(I^{\prime },L\right)=\sum_{I\in 𝒥}\frac{⊕I,L}{\left|𝒥\right|}\right]$. This is the Bayesian probability model.

In order to modify ⊕(*I*′, *L*) posteriori after observing the voting of the system users, it is necessary to model the system users. In this manuscript, the user model of the adaptive intelligent recommendation system applies two parameters to represent the accuracy of T and F that the system users can detect, which are called the sensitivity and specificity of the system users. Because of the sparsity of labels in the system user set and archive sets in this manuscript, the specificity of the system users is much higher than the sensitivity of the system users. In addition, using two parameters instead of a single shared parameter to represent the accuracy of the system users greatly improves the identification level of classification labels in this probability model.

If the adaptive intelligent recommendation system users with the sensitivity *p*_*tp*_ and specificity *p*_*tn*_ think a label = yes, then the prior value can be multiplied by the likelihood ratio [*p*_*tp*_+(1−*p*_*tn*_)]/[(1−*p*_*tp*_)+*p*_*tn*_] to correct the posterior. In this model, the probability value of ⊕(*I*, *L*) is known by the system, such errors can be reduced if the utility model is associated with different costs of voting classification.

**FIGURE 3:**
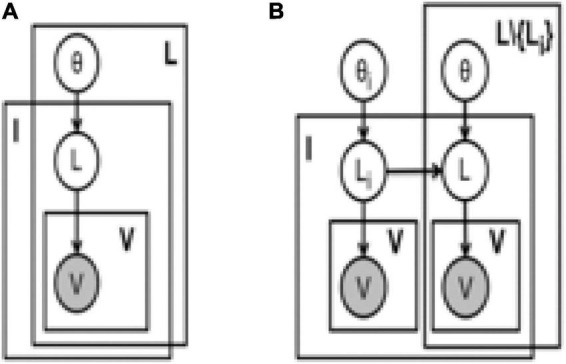
Generation probability model of multi-label classification. **(A)** Independent. **(B)** MLNB.

Assume that the labels are independent, as shown in the graphical model in Figure 3A. If the label set is represented by ℒ, then the independent model has (|ℒ| + 2) parameters, corresponding to each label of the label’s prior probability and all the system user models assumed in this manuscript. The marginal label probability is *P*(*L*|*v*)∝*P*(*L*)*P*(*v*_*L*_|*L*), where *L* ∈ ℒ is the Boolean random variable corresponding to the label result ⊕(*I*′, *L*) of an archive document, *v*_*L*_⊆*v* is the number of observed votes associated with the result. This independent model is called the benchmark probability model.

In this manuscript, expectation maximization (EM) (Dubois-Lacoste et al., 2017; Millar, 2017) and probability model parameters are used to estimate the values of these potential labels. By assuming a weak symmetric β prior and calculating the maximum posteriori estimation, the problems that may occur at the beginning of the classification step are avoided, and the Bayesian estimation of the parameter value is obtained.

### Label co-occurrence modelling

Assume that the labels generated through the above model are independent. Because the archives classified as “personnel” are more likely to be archives in the promotion and transferring categories than archives in the preventive category, it is necessary to learn the label joint probability model. In this model, when a system user knows that an archive document belongs to a certain category, the posteriori of all other categories can be modified. This modification will also affect the label with the highest information value determined by the control strategy proposed in this manuscript.

In this model, *I*, *L*, and *V* correspond to archive documents, labels and votes, respectively. The multi-label naive Bayesian model in Figure 3B is used to predict the generation probability of the label *L_i_* and there are |ℒ| such models.

In this manuscript we explore a simple model called weighted multi-label naive Bayes (WMLNB) (Chilton and Schäffner, 2002; Yapp et al., 2020). For each label in this model, we construct a directed star map from that label to all other labels; the graph model in Figure 3B shows the directed star map of label *L_i_*. With the independent model concept defined in this manuscript, the marginal label probability of MLNB model is


$$P\left(L|v\right)\propto P\left(L\right)P\left(v_{L}|L\right)\prod_{L^{\prime }\in ℒ\,\left\{L\right\}}\sum_{L^{\prime }}P\left(L^{\prime }|L\right)P\left(v_{L^{\prime }}|L^{\prime }\right)$$

To calculate the marginal probability of all labels, it is necessary to calculate *O*(|ℒ|^2^) for each archive document, including the potential label variables in the graphic model. To estimate the parameters of WMLNB model, it is necessary to reuse the parameters and label predictions obtained by EM operation on the independent models. These predictions make the conditional label probability *P*(*L*′|*L*) of the supplementary 2(|ℒ^2^|−|ℒ|) approximate to the expected value of the archives with both label *L* and label *L*′.

### Select questions to ask

The distributed intelligent recommendation system adopts a simple circular strategy, while the adaptive system in this manuscript uses greedy strategy to retrieve labels, so that the system users’ voting provides the maximum information value for these labels. In other words, each time the adaptive method requires the system users to provide new votes, it is to select a group of votes that can minimize the label prediction uncertainty. Information theory provides a standard to measure the uncertainty of label prediction distribution. In the joint entropy *H*(ℒ) = −∑_*l* ∈ *dom*ℒ_*P*(*l*)*log*(*P*(*l*)), the domain ℒ contains all possible assignments to the variables in the domain, and *l* is one of them. Let 𝒜⊂𝒱, where 𝒱 denotes an infinite set of possible future votes. After set 𝒜 obtains the votes, the expected uncertainty of label prediction distribution is the conditional entropy *H*(ℒ|𝒜) = $-\sum {}_{\begin{array}{c} I\;\in \;dom\;ℒ\\ a\;\in \;dom\text{ 𝒜} \end{array}}$ *P*(*l*, *a*)*logP*(*l*|*a*). It is called expected information gain, or mutual information *I*(ℒ;𝒜)=*H*(ℒ)−*H*(ℒ|𝒜). Because of the relevance of the issue, it is difficult to calculate the intelligent combination 𝒜 of information gain maximization. The research of Nemhauser, Wolsey and Fisher shows that the greedy algorithm provides a solution within the intelligent value of (1−1/*e*)≈63% (Nemhauser et al., 1978; Millar, 2017). Krause and Guestrin gave a greedy algorithm for the approximate intelligent variable quantum subset, and proved that there is no upper bound unless P = NP (Nemhauser et al., 1978).

The greedy algorithm uses greedy heuristic algorithm (Leskovec et al., 2007; Lin et al., 2020) to add one vote at a time for V ∈ 𝒱, and obtains a set of future voting set 𝒜. In order to improve the heuristic algorithm, the conditional independence assumption is added to the model, assuming that H(V|ℒ) is simplified to local conditional entropy *H*(*V*|*L*_*V*_), where *L*_*V*_ ∈ ℒ is the label corresponding to vote *V*.

#### Theorem 2

Let every vote in V ∈ 𝒱 be independent of all other votes labelled as L_V_. Let 𝒜 be the future voting set accumulated so far by greedy algorithm, V_L_ represents any future vote of label L, then set 𝒜 consists of future votes V* continuously added, V* ∈ *argmax*_*L* ∈ ℒ_*H*(*V*_*L*_|𝒜)−*H*(*V*_*L*_|*L*) is in the intelligent value range (1−1/e).

Applying the research results of Krause and Guestrin (Yan et al., 2016) to this model, we can prove the above conclusion. When the greedy algorithm chooses the first vote, 𝒜 is initially empty, so H(V|𝒜) is *H*(V). This manuscript uses this greedy strategy and WMLNB model for label co-occurrence to optimize the classification process.

## Experiments

The paper tries to compare various strategies in classification control. This manuscript first analyses the number of votes saved (lossless, one-way) by each strategy, and the number of votes saved when setting the threshold value *T*={2,3,4}, as well as the performance of the classification generated, and then compares the improvement of threshold voting. Next, this manuscript evaluates the prediction performance of the probability model and compares it with the initial strategy of the distributed intelligent recommendation system.

### Data [21.22] sets

In order to better analyse the effect of different classification algorithms, this manuscript controls the different forms of label elucidation strategy, and selects a group of fixed candidate classification labels. Specifically, this manuscript removes labels with low probability, and produces 33 manageable labels, such as upward, parallel, downward archive documents; personnel, general affairs, preventive, taxation, secretary, notice; Circular, S/O Circular, Despatches, P/N, S/O Letter; economy, trade report, diplomacy, exhibition, clothing, intelligence, and so on. A random subset of 100 archive documents is constructed by classifying archive documents for each label.

In this manuscript, the voting process of the adaptive intelligent recommendation system users simulates the process of “classifying” the 100 archive documents and 33 labels in the distributed intelligent recommendation system. In this manuscript, we collected the votes of *k*=15 adaptive system users for the seven classification labels of each archive document. The system ensures that the system users select at least one label through the reward mechanism, or indicates that the displayed labels are not applicable to the archive document. The purpose of collecting these data is to compare different control strategies and control system users’ errors, because each control strategy will see the same system users’ responses.

The table shows the F score, the number of votes per archive document, and the percentage of votes saved when colleting 5 votes for each label by the adaptive method compared with that by the distributed intelligent recommendation system.

### Threshold method

The first experiment compares the threshold voting correction with the original threshold voting implemented in the distributed intelligent recommendation system. Because the distributed intelligent recommendation system uses different threshold settings in adaptive context filtering, this manuscript tests the five total votes when the threshold is *T*={2,3,4}. Table 1 shows the number of votes obtained by using lossless stop method and one-way heuristic method for each archive document, as well as the number of votes saved compared with the original method in the distributed intelligent recommendation system (33 × 5 = 165 votes per archive document). When *T*=4, lossless stop can save up to 58% of votes, which is exactly the same as the threshold voting process in distributed intelligent recommendation system.

**TABLE 1:** Comparison of threshold voting methods.

Method	*T*	F score	Votes	Votes saved (%)
Lossless	2	0.83	105	26
One-way	2	0.82	96	42
Lossless	3	0.84	102	38
One-way	3	0.83	69	58
Lossless	4	0.75	70	58
One-way	4	0.70	38	77

**FIGURE 4:**
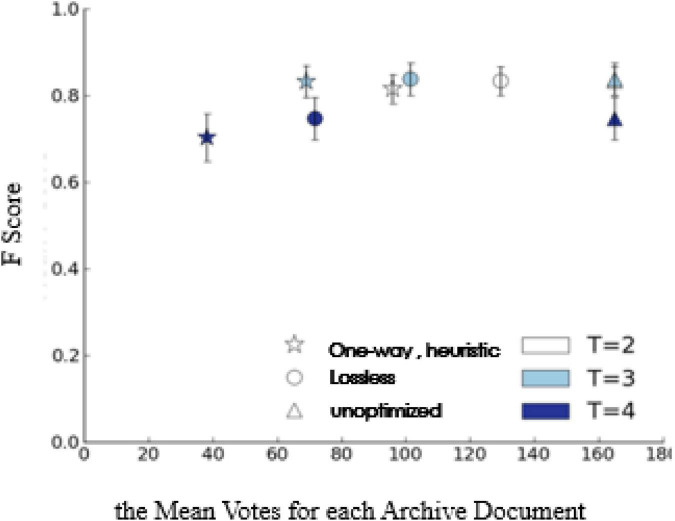
F score vs. cost of threshold voting improvement.

In order to better understand the impact of one-way heuristic method on classification performance, in Figure 4, we draw the relationship between F-means and the number of votes by the lossless stop and one-way heuristic method. When the threshold *T*={2,3}, the one-way strategy can significantly reduce the number of votes without introducing the statistically significantly reduced F-means. When *T*=4, the decrease of F-means is statistically significant (two tailed paired *t*-test, *p* < 0.01). If the first vote is negative, then the one-way heuristic under this threshold setting returns F. In this situation, the sensitivity of the system users is significantly lower than their specificity, which is sub-intelligent.

**FIGURE 5:**
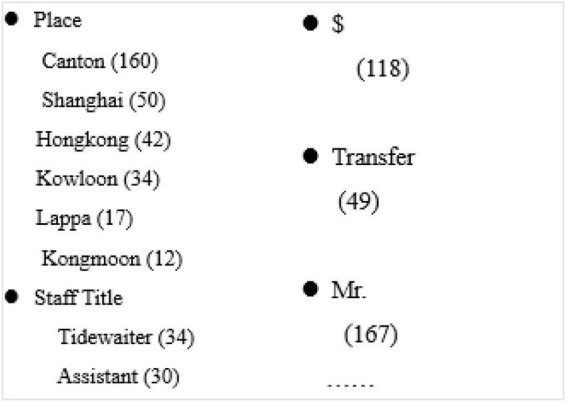
The performance of the one-way strategy when threshold = 3.

In Figure 5, when the threshold *T*=3, the one-way strategy can produce good classification results (excerpts are shown in the figure) by only using 42% of the labour force required by the distributed intelligent recommendation system.

In addition to classification performance, this manuscript is also interested in how the improved control strategy affects the classification quality of the final output archives translated. When one-way heuristic method is applied, visual inspection of errors in the output classification does not show any quality degradation. Figure 5 shows the high-quality classification generated by the one-way heuristic method with the threshold *T*=3.

### Reasoning-based methods

In order to prove the effectiveness of the reasoning method, this manuscript collects votes from the adaptive intelligent recommendation system to compare the performance of various reasoning and control strategies, and applies multi-label classification and classification construction to the case of large-scale archives.

This manuscript tests three reasoning methods (MLNB, independence and majority) and two control strategies (greedy and circular). MLNB and the independent reasoning method are described in the previous section. The majority strategy performs the simple majority voting evaluation with the default negative answers. Greedy control strategy uses the heuristic method from Theorem 2 to select labels that maximize information gain, while the circular strategy votes layer by layer.

In order to test the performance of this model when the number of archive tasks increases, this manuscript sets aside one archive document from 100 archive documents for cross validation to evaluate the performance of this model, estimates the model parameters with 99 archive documents, and conducts five votes for each archive-label pair in the training set.

**FIGURE 6:**
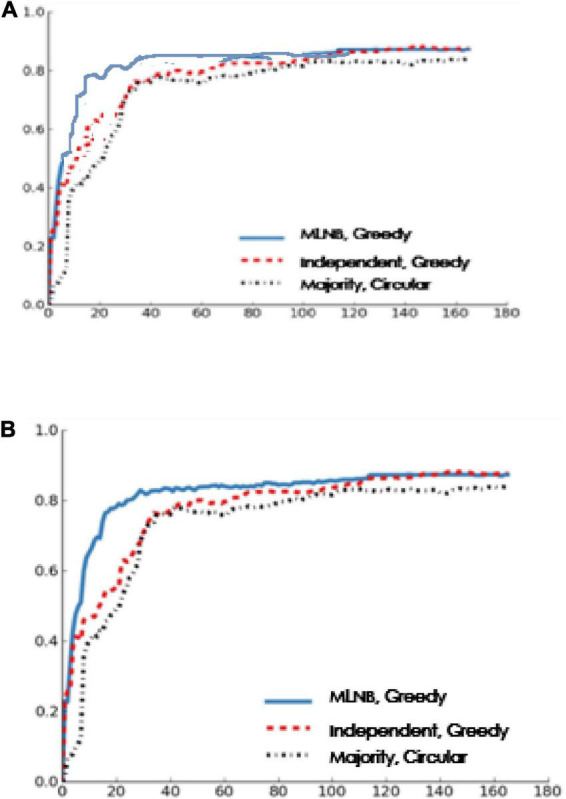
**(A)** Performance and votes when *T* = 2. **(B)** Performance and votes when *T* = 3.

Figure 6 shows the results of this experiment. MLNB and the independent strategy are obviously better than the simple Circular strategy, and MLNB in particular achieves a high performance level soon. In the first 47 votes, the performance advantage of MLNB over the independent strategy is statistically significant at the 0.05 significant level (using two tailed paired *t*-test), which verifies the hypothesis of this manuscript.

In this archive set, the voting strategy of distributed intelligent recommendation system (if four fifths of the system users think it is applicable, they will accept a label) requires 165 system users to vote for each archive document. Compared with the intelligent data, the F-score is 75%. In contrast, for the one-way strategy, when the threshold *T*=2,T=3 the F-score is 88% and F-score is 83%, and only 45 and 46% of the system users are employed. The greedy control strategy MLNB applied in this manuscript obtains 76% F-score after only 16 users vote for each archive document, and the number of the required system users is less than 10% of the number of users required by the distributed intelligent recommendation system to achieve similar performance.

### Batch label processing control strategy

To apply the control strategy in the adaptive allocation system, it is necessary to combine the archive documents together, so that a system user can answer multiple questions about an archive document at the same time; see the example in Figure 1. Theorem 2 provides a method to select batch labels, collecting a group of votes by using the greedy heuristic algorithm. The control strategy *k* selects only the first *k* labels sorted by the greedy heuristic method before collecting votes, which is called proximity algorithm.

**FIGURE 7:**
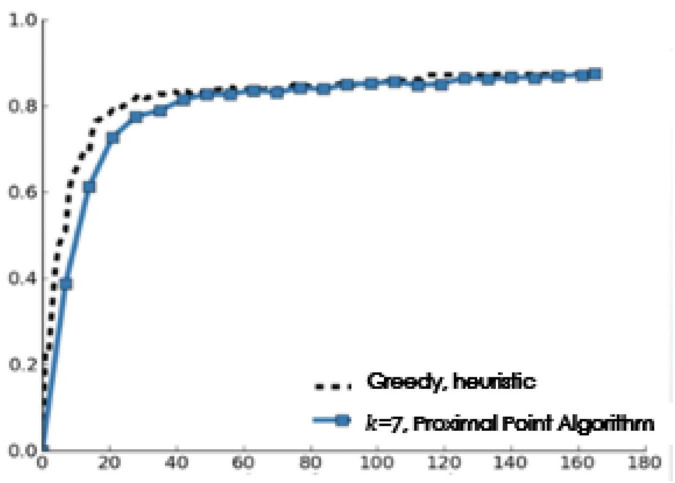
Performance and votes when *k*=7.

Figure 7 shows the performance and the number of votes of batch labels when *k*=7 (compared with the MLNB algorithm for selecting a single label).

The experiments show that the proximity algorithm achieves the best balance between classification performance and calculation complexity. Figure 7 shows that when *k*=7 (the same number as that in the distributed intelligent recommendation system and that in the field test in this manuscript), compared with the MLNB with a single label, the performance of the MLNB with proximity algorithm is slightly reduced. This difference was statistically significant (at the 0.05 significance level, using the two tailed paired *t*-test). When there are about 35 votes per archive document, the batch label selection of MLNB is better than the independent strategy of single label selection.

In terms of performance gain, the cumulative greedy method is not as good as the proximity algorithm. The cumulative method can’t improve its performance, which may be due to the fact that the labels in a batch must be different in this manuscript (asking the same system user the same question many times will not bring benefits). Considering this setting, the proximity algorithm is an effective heuristic method, and the cost does not increase compared with the single label selection.

**FIGURE 8:**
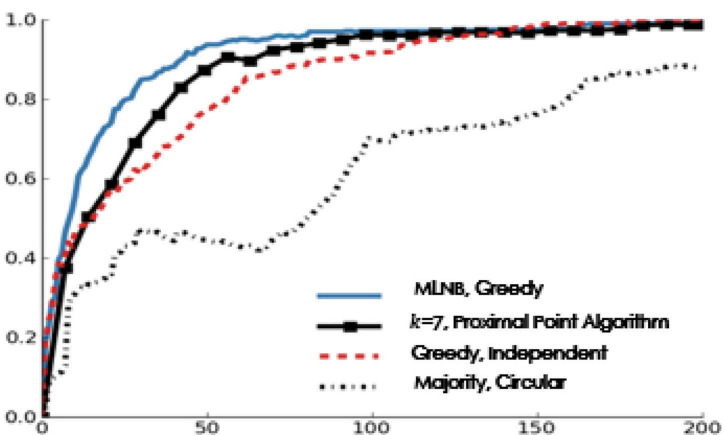
Performance and votes when sensitivity = 0.6 and specificity = 0.8.

Figure 8 shows the relationship between the performance and the number of votes for the more difficult simulation archive tasks (sensitivity = 0.6, specificity = 0.8).

### Simulation experiment

Considering the skill levels of the system users, the intelligent control program must be stable. In order to evaluate the performance of our method on these classification problems, we simulate the system users with sensitivity of 60% and specificity of 80%. In this manuscript, we use archive-label pair to do this experiment in pure simulation environment. In the data set of this manuscript, assuming that the average sensitivity and specificity of the system users are 76 and 98%, respectively, in Figure 8, although the ability of system users is lower, the final overall performance is higher, which may be attributed to the difference between the best answer provided by task issuers and the voting decision made by system users on the adaptive intelligent recommendation system. Figure 8 shows the same model ranking with statistical significance as seen in the real system user voting, which shows that the results of this manuscript are applicable to a wide range of multi-label classification tasks.

## Conclusion

Machine learning and decision-making theory greatly reduce the labour force required by the adaptive allocation system. However, so far, most of the work has focused on optimizing relatively simple workflow, such as iterating to improve workflow. Classification generation and construction is an important task, which requires complex workflow to create global consistent interpretation for large-scale data sets from small-scale data system users. Although the previous classification and construction of distributed intelligent recommendation system has a bright future, it has become the object of decision-making theory optimization due to too much labour force consumption.

The adaptive algorithm investigated in this manuscript is an improvement of the distributed intelligent recommendation system algorithm, which adopts a new method to solve the problem of label elucidation and multi-label classification. For the former, this manuscript constructs the Polya urn combination model, which allows the calculation of the relative cost of stopping the label generation stage in advance. For the archive classification problem with relatively fixed label set, this manuscript proposes four models: lossless, one-way, Bayesian probability model and MLNB model with label co-occurrence. The latter two models support greedy control strategy, that is, to select the label with the largest amount of information in the next intelligent label constant factor, so that users can evaluate it. This manuscript also provides a batch processing strategy, which makes the multi-label classification method of this manuscript highly universal and practical.

In this manuscript, the relative effectiveness of the multi-label classification method is evaluated through the field experiment on the adaptive intelligent recommendation system. The voting strategy of the distributed intelligent recommendation system requires 165 system users to vote for each archive document. The adaptive method proposed in this manuscript can achieve better performance with fewer users. Especially when only 16 adaptive system users vote for each archive document or the number of the adaptive system users is less than 10% of the users required by the distributed intelligent recommendation system, the performance of the adaptive method of the greedy control strategy MLNB is better than that of the distributed intelligent recommendation system.

Experiments show that when the answer to a test question can provide information about other archives to be translated, the adaptive intelligent recommendation system needs to give priority to this question. When there are many candidate questions, the sub-module optimization method can be used to help the system calculate the next test question efficiently. The system can model the system users and improve their performance without the help of task issuers; in this system, a small amount of training data combined with probability model can generate significantly better strategies.

## Data availability statement

The original contributions presented in this study are included in the article/supplementary material, further inquiries can be directed to the corresponding author.

## Author contributions

CY supervised and revised the manuscript. Both authors are involved in the data collection, experiments, and analysis of the experiment results.

## References

[B1] Chilton L. B. Little G. Edge D. Weld D. S. Landay J. A. (2013). “Cascade: Crowdsourcing taxonomy creation,” in *Proceedings of the SIGCHI conference on human factors in computing systems, CHI’13* (Paris: ACM), 1999–2008. 10.1145/2470654.2466265

[B2] Chilton P. Schäffner C. (eds) (2002). *Politics as text and talk: Analytic approaches to political discourse*, Vol. 4. Amsterdam: John Benjamins Publishing. 10.1075/dapsac.4 33486653

[B3] Dubois-Lacoste J. Pagnozzi F. Stützle T. (2017). An iterated greedy algorithm with optimization of partial solutions for the makespan permutation flowshop problem. *Comput. Oper. Res.* 81 160–166. 10.1016/j.cor.2016.12.021

[B4] Krause A. Guestrin C. (2005). “Near- intelligent nonmyopic value of information in graphical models,” in *Proceedings of the 21st conference in uncertainty in artificial intelligence, UAI ‘05* (Edinburgh: AUAI Press), 324–331.

[B5] Leong W.-F. Yen G. G. (2008). PSO-based multi-objective optimization with dynamic population size and adaptive local archives. *IEEE Trans. Syst Man Cybern. B Cybern.* 38 1270–1293. 10.1109/TSMCB.2008.925757 18784011

[B6] Leskovec J. Krause A. Guestrin C. Faloutsos C. VanBriesen J. Glance N. (2007). “Cost-effective outbreak detection in networks,” in *Proceedings of the 13th ACM SIGKDD international conference on knowledge discovery and data mining* (New York, NY: Association for Computing Machinery), 420–429. 10.1145/1281192.1281239

[B7] Lilan C. Yongsheng C. (2021). Spatial-temporal adaptive intelligent allocation of archival tasks. *IEEE Access* 9 25809–25817. 10.1109/ACCESS.2021.3057362

[B8] Lin Q. Wenming C. He Z. He Z. (2020). Mask cross-modal hashing networks. *IEEE Trans. Multimed.* 23 550–558. 10.1109/TMM.2020.2984081

[B9] Lo J. C. Fujiwara E. (1996). Probability to achieve TSC goal. *IEEE Trans. Comput.* 45 450–460. 10.1109/12.494102

[B10] Mahmoud H. (2008). *Pólya urn models.* London: Chapman and Hall/CRC. 10.1201/9781420059847

[B11] Millar L. A. (2017). *Archives: Principles and practices.* London: Facet Publishing. 10.29085/9781783302086

[B12] Nemhauser G. L. Wolsey L. A. Fisher M. L. (1978). An analysis of approximations for maximizing submodular set functions—I. *Math. Program.* 14 265–294. 10.1007/BF01588971

[B13] Paul G. Harrison M. (2005). Allocation under dictatorship: Research in Stalin’s archives. *J. Econ. Lit.* 43 721–761. 10.1257/002205105774431225

[B14] Research Square (2021). *The optimal allocation policy via multi-label tagging for translation tasks of China’s imperial maritime customs archives*. Available online at: https://assets.researchsquare.com/files/rs-887204/v1_covered.pdf?c=1632153615 (accessed 2019).

[B15] Ross S. M. (2014). *Introduction to probability models[M].* Cambridge, MA: Academic press. 10.1016/B978-0-12-407948-9.00001-3

[B16] Sun W. (2017). “Accurate EM simulation of SMT components in RF designs,” in *Proceedings of the 2017 IEEE radio frequency integrated circuits symposium (RFIC)* (Honolulu, HI: IEEE), 140–143. 10.1109/RFIC.2017.7969037

[B17] Wang R. Shen M. Wang T. Cao W. (2019). L1-norm minimization for multi-dimensional signals based on geometric algebra. *Adv. Appl. Clifford Algebras* 29:33. 10.1007/s00006-019-0950-7

[B18] Wang R. Shen M. Wang X. Cao W. (2021). RGA-CNNs: Convolutional neural networks based on reduced geometric algebra. *Sci. China Inf. Sci.* 64:129101. 10.1007/s11432-018-1513-5

[B19] Yan X. Wu Q. Sheng V. S. (2016). A double weighted Naive Bayes with niching cultural algorithm for multi-label classification. *Int. J. Pattern Recognit. Artif. Intell.* 30:1650013. 10.1142/S0218001416500130

[B20] Yapp E. K. Li X. Lu W. F. Tan P. S. (2020). Comparison of base classifiers for multi-label learning. *Neurocomputing* 394 51–60. 10.1016/j.neucom.2020.01.102

